# Economic value of diastasis repair with the use of mesh compared to no intervention in Italy

**DOI:** 10.1007/s10198-024-01685-z

**Published:** 2024-03-14

**Authors:** Carla Rognoni, Alessandro Carrara, Micaela Piccoli, Vincenzo Trapani, Nereo Vettoretto, Giorgio Soliani, Rosanna Tarricone

**Affiliations:** 1 Centre for Research on Health and Social Care Management (CERGAS), SDA Bocconi School of Management, Bocconi University, Milan, Italy; 2 First General Surgery Unit, Ospedale Santa Chiara, Trento, Italy; 3 UOC di Chirurgia Generale, d’Urgenza e Nuove Tecnologie dell’OCB (Ospedale Civile di Baggiovara), AOU (Azienda Ospedaliero Universitaria) di Modena, Modena, Italy; 4 UOC di Chirurgia Generale del Presidio Ospedaliero di Montichiari, AO Spedali Civili di Brescia, Brescia, Italy; 5 Azienda Ospedaliero Universitaria, Ferrara, Italy; 6 Department of Social and Political Sciences, Bocconi University, Milan, Italy

**Keywords:** Rectus abdominal diastasis, RAD, Abdominoplasty, Cost-effectiveness, Budget impact, Economic evaluation, Synthetic mesh, Biosynthetic mesh, NHS, Society

## Abstract

**Aim:**

Rectus abdominal diastasis (RAD) can cause mainly incontinence and lower-back pain. Despite its high incidence, there is no consensus regarding surgical indication. We aimed at comparing RAD repair (minimally invasive technique with mesh implant) with no treatment (standard of care – SOC) through cost-effectiveness and budget impact analyses from both National Healthcare Service (NHS) and societal perspectives in Italy.

**Methods:**

A model was developed including social costs and productivity losses derived by the online administration of a socio-economic questionnaire, including the EuroQol for the assessment of quality of life. Costs for the NHS were based on reimbursement tariffs.

**Results:**

Over a lifetime horizon, estimated costs were 64,115€ for SOC and 46,541€ for RAD repair in the societal perspective; QALYs were 19.55 and 25.75 for the two groups, respectively. Considering the NHS perspective, RAD repair showed an additional cost per patient of 5,104€ compared to SOC, leading to an ICUR of 824€. RAD repair may be either cost-saving or cost-effective compared to SOC depending on the perspective considered. Considering a current scenario of 100% SOC, an increased diffusion of RAD repair from 2 to 10% in the next 5 years would lead to an incremental cost of 184,147,624€ for the whole society (87% borne by the NHS) and to incremental 16,155 QALYs.

**Conclusion:**

In light of the lack of economic evaluations for minimally invasive RAD repair, the present study provides relevant clinical and economic evidence to help improving the decision-making process and allocating scarce resources between competing ends.

**Supplementary Information:**

The online version contains supplementary material available at 10.1007/s10198-024-01685-z.

## Introduction

Diastasis recti or rectus abdominal diastasis (RAD) is an underestimated but extremely common disorder, which is characterized by an excessive separation of the right side from the left side of the rectus abdominis muscle (enlargement of the linea alba) which occurs in 20–30% of women after pregnancies, or in a lower rate in men, mostly after an important weight gain. Diastasis is a physiological, normal process after childbirth as long as it resolves within 12 months. Conversely, it can cause abdominal swelling, dyspepsia, low back pain, urinary stress incontinence back pain, incontinence and abdominal pain [1, 2]. In addition, it can result to an unsatisfactory body image, which together with the aforementioned functional symptoms, might lead to a significant negative impact on patients’ quality of life. It is estimated that one out of three women after pregnancy and one out of two after 50 years of age would present with RAD [3, 4].

A recent study [5], which evaluated the quality of life of women postpartum through a numerical scale (0–10) and a hernia specific survey (HerQLes), assessed that at 3–6 months postpartum, quality of life significantly improved for women without diastasis compared with women with moderate or severe diastasis. Moreover, the aesthetic discomfort felt by patients was significantly increased by the presence of diastasis on a numerical scale at 3–6 months (4.2 ± 2.9 for women without diastasis vs. 5.3 ± 2.8 for women with diastasis, *p* = 0.03). Among the problems, urinary incontinence is accompanied by high levels of stress and embarrassment owing to discomfort arising from urine leakage. Moreover, incontinence may seriously affect normal social interaction and leisure activities among affected individuals. This effect is not only physiological, but can also have a great impact on the patient’s psychological health [6]. Data on an Italian population [7] showed that the incidence of depression was 11% in people with urinary incontinence and 7% in those without this condition.

A prospective observational study of 110 consecutive patients affected by midline primary hernias and RAD showed that endo-laparoscopic reconstruction of the abdominal wall with mesh positioning was a feasible, safe and effective procedure [8]. After a mean follow-up of 14 months, the morbidity rate was 9.1% and no recurrences were recorded. Data regarding the impact of surgery on patients’ quality of life (EuraHSQol), on chronic low back pain (Oswestry Disability Index, ODI) and urinary stress incontinence (Incontinence Severity Index, ISI) showed improvements in 93%, 77%, and 63% of the cases, respectively.

Despite the high incidence of this disease, still there is no consensus regarding surgical intervention.

In Italy, RAD repair may be reimbursed by the NHS when the intervention is combined with umbilical hernia repair; in these cases, the DRG 160 (hernia procedures except inguinal & femoral, age > 17, without complications) is usually applied, with a reimbursement tariff at national level of 1,371€. In case complications occur during the surgical intervention, the considered DRG is 159 (hernia procedures except inguinal & femoral, age > 17, with complications), with a national reimbursement tariff of 4,892€.

Although advantages have been demonstrated from the clinical point of view and for patients’ quality of life, there is insufficient evidence to determine the cost-effectiveness of treatments for diastasis recti in women. A recent systematic literature review [9] reported that the cost-effectiveness of treatments for RAD could not be estimated due to the lack of evidence on treatment effects.

The aim of the present paper is to develop knowledge about the clinical and economic implications that can support the decision-making process when the management of patients with RAD is at stake. In particular, the objective of the study was to compare RAD repair by mini-invasive technique (with mesh implant), with no treatment through the collection of real-world data [10] and to develop models in order to carry out a Cost-Effectiveness Analysis (CEA) and a Budget Impact Analysis (BIA) from both societal and NHS perspectives. The Italian Programme for HTA of Medical Devices considers as relevant both these perspectives when assessing the introduction of innovative devices [11]. The CEA will provide the cost-effectiveness profile of RAD repair to help support the adoption of this innovative technology at national level, while the BIA will show the financial impact of the introduction and adoption of this technology in the clinical practice.

## Methods

The study has been approved by Bocconi Ethics Committee (code FA000428, approval date 15 June 2022).

### Data collection

In order to populate the economic evaluation models, we considered three sources of data: (1) the “Italian Hernia Club” registry, (2) an ad hoc developed socio-economic questionnaire and (3) a questionnaire for the evaluation of patients’ quality of life. The sources are described in detail in the following paragraphs.

#### “Italian Hernia Club” registry

The “Italian Hernia Club” is an observational, prospective, multicenter registry that began collecting data through electronic medical records accessible via the web in 2015 [12, 13]. Ten Italian clinical centers, geographically distributed in the country, started data collection on adult patients undergoing complex abdominal hernia repair with biosynthetic prostheses. The registry has been subsequently extended, also internationally, to collect data (i) on hiatal hernia repair, (ii) on the use of biosynthetic meshes for hernia prevention in patients undergoing intestinal stoma closure and (iii) on concomitant hernia and RAD repair. The registry records all the data related to the preintervention visit, the intervention itself and the follow-up visits (predefined timing at 8 days, 30 days, 6–12–18–24–36–48–60 months). In cases of suspected relapse or complications, telephone follow-up is associated with an outpatient visit; if necessary, imaging examinations (ultrasound, CT scan) and blood tests are performed.

Currently the registry contains data on 259 women with mean age of 41 years who underwent intervention for repair of hernia and rectus diastasis with mesh implant (77% synthetic, 23% biosynthetic). The data derive from 7 clinical centers, six geographically distributed in Italy (148 patients from APSS Trentino, Trento; 54 patients from ASST Spedali Civili di Brescia, Brescia; 19 patients from AO S. Anna, Ferrara; 5 patients from OC S. Agostino-Estense, Baggiovara-Modena; 2 patients from AOU Cagliari and 5 patients from AOU Ascoli Piceno) and one in Brazil (26 patients from Hospital das Clinicas, Sao Paulo). All operators performed the technique following the same steps and using the same materials and tools.

Regarding RAD repair, so far, the registry has collected demographic data and data on the main complications (hematoma, seroma, posterior rectus sheaths disruption, surgical site infection) and recurrences with a maximum follow-up of 24 months. Rates of complications at the different follow-ups have been analyzed and included in the model.

#### Socio-economic questionnaire

The consumption of health and non-healthcare resources that are not routinely collected by hospitals, such as out-of-pocket resources and productivity losses of adult women with RAD, has been estimated through a socio-economic questionnaire purposely developed and validated by the clinicians participating in the study. Inclusion criteria for the patients’ enrollment were:

- Patients able to provide informed consent for participation;
- Adult women (age > = 18 years);
- Patients with a diagnosis of RAD.

Patients not satisfying inclusion criteria were excluded from the analysis.

The questionnaire included different sections (see Appendix 1 for full questionnaire):

1. Informed consent and introduction to the aims of the questionnaire;
2. Personal data (age, occupational status) and possible information on RAD repair (if performed, type of intervention and date of intervention);
3. Identification and management of main RAD complications like incontinence and lower back pain: past and periodical expenses (out-of-pocket costs) for sanitary towels, physiotherapy/exercises, painkillers or other;
4. Number of control visits/examinations in the last 3 months, mean working time lost per visit/examination, mean out-of-pocket cost per visit/examination (ticket or expense if performed privately, expense for transportation, meals away from home);
5. Need of assistance in everyday life: paid assistance in the past month;
6. Productivity losses: days of work lost in the past 3 months for problems related to RAD.

#### Questionnaire for the evaluation of patients’ quality of life

Data on quality of life has been collected via the EuroQol 5D-5L questionnaire, which is a standardized tool that allows measurement of the health status of respondents and their quality of life (https://euroqol.org/eq-5d-instruments/eq-5d-5 L-about/) [14]. An algorithm allows the calculation of a final score (utility coefficient u, between 0 and 1) based on the attribution of weights for each questionnaire answer. The higher the score, the better the health.

Inclusion criteria were the same as for the socio-economic questionnaire.

#### Questionnaires implementation and administration

The socio-economic questionnaire and the EuroQol have been implemented through Qualtrics platform and administered online. Informed consent form is presented to the respondent before questionnaires administration; the respondent is asked to give the informed consent by clicking a dedicated single choice field (I give informed consent/I do not give informed consent); in case the respondent accepts, she visualizes the questionnaires section, otherwise no questionnaires is presented and the survey is closed. Online questionnaires do not collect data able to retrieve the identity of the respondents.

In order to minimize input errors, coded fields have been applied where possible. When a text input was requested, controls on data type (e.g., only numbers) were possibly applied.

In order to reach women with RAD, an anonymous link to the questionnaire has been shared with an Italian association of about 30,000 patients (Diastasi Donna, http://www.diastasidonna.it/). Data collection started in August 2022 and ended on 10th September 2022. No minimum sample size was requested.

### Cost-effectiveness analysis (CEA) model

The implementation of a cost-effectiveness Markov model aimed to compare RAD repair with mesh implant versus no intervention from both NHS and societal perspectives in Italy. The analysis was reported according to Consolidated Health Economic Evaluation Reporting Standards (CHEERS) [15, 16]. The CHEERS checklist is reported in the electronic supplementary material (Appendix 2).

**Fig. 1:**
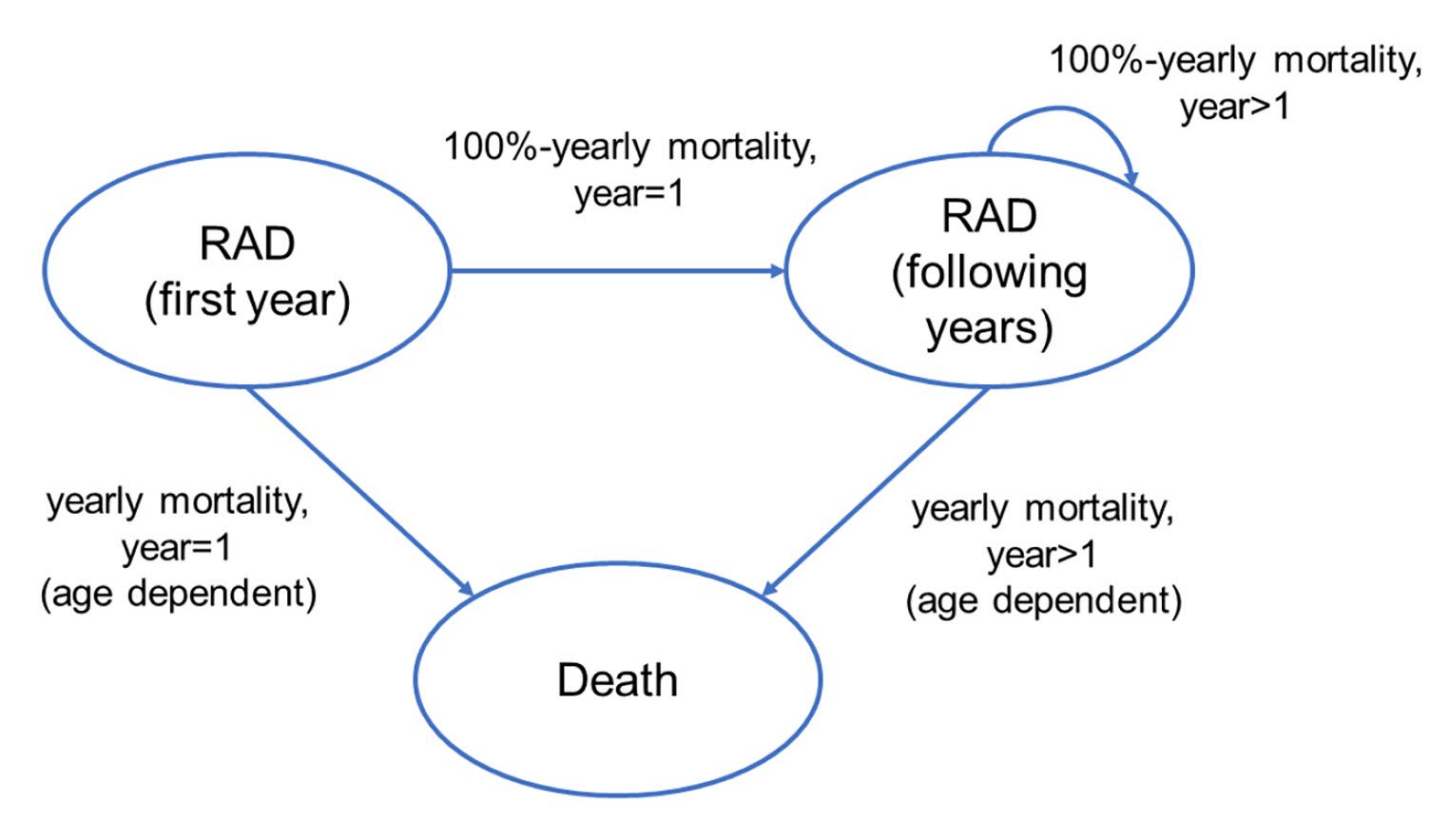
Markov model representation

The model, which considered women with RAD with a mean age of 43 years, as estimated from the analyzed sample (see Results section for details on data collected), projected costs and QALYs on a lifetime horizon in order to evaluate the incremental cost-utility ratio (ICUR). The model (Fig. 1) considered three health states, “RAD (first year)”, “RAD (following years)” and death. A Markov cycle of 1 year has been applied. The process starts in the “RAD (first year)” health state. This distinction was particularly useful to represent the pathway of surgical patients, for whom we distinguished costs and QALYs in the first year (FUP < = 1 year) from the subsequent years (FUP > 1 year) given that, in the post-surgical period, costs may be higher due to contemporary activities like physiotherapy or postural training and quality of life may be low due to surgical consequences. After one year, all patients move to “RAD (following years)” health state. The strategy considering no intervention didn’t distinguish costs and health outcomes between first and subsequent years, therefore yearly costs were the same for both health states. Death state represents general mortality for Italian females [17]. Transition probabilities to death are reported in Supplementary Table 1.

The ICUR was calculated as the difference in the mean expected costs divided by the difference in the mean expected QALYs of the considered strategies. The paucity of information on the appropriate way of estimating the WTP threshold and the lack of a universally adopted value do not facilitate the interpretation of cost-effectiveness studies results. Around the world threshold values range from 15,000€ to 80,000€ [18, 19]. In Italy, applied thresholds are in the range 25,000–74,700€ [20–22]. In the context of the present analysis an intermediate value of 50,000€/QALY has been considered.

A discount rate of 3% has been applied to QALYs and costs [23]. Costs were derived from the responses to the socio-economic questionnaire, while utilities used to estimate QALYs were derived from the responses to EuroQol questionnaire.

Both probabilistic and one-way sensitivity analyses have been performed to evaluate the robustness of the model results. For the former, 10,000 Monte Carlo simulations have been performed by extracting parameters from distributions (gamma for costs and beta for utilities/percentages). In case the sources referencing the parameters reported standard deviations, these ones were applied to estimate parameters variations, otherwise a variation of ± 20% of the baseline values has been used. The same variation ranges have been applied for one-way sensitivity analyses. Results of PSA have been reported as points in the cost-effectiveness plane and as acceptability curves. Tornado diagrams were obtained for the representation of deterministic analyses.

In addition to the conventional presentation of CEA results, RAD repair and SOC have been compared through incremental net monetary benefit (INMB) metric, which is calculated as the difference between the benefits and the costs of each strategy, expressed in monetary terms. Monetary valuations of benefits are generally obtained through the application of a willingness to pay (WTP) threshold. In this specific case a conservative threshold of 25,000€/QALY has been applied [20]. A positive INMB would indicate that RAD repair is a cost-effective strategy compared with SOC at the given WTP threshold. In this case, the cost to obtain the benefit would be less than the maximum amount that the decision-maker would be willing to pay for this benefit. This type of analysis is a rational approach for economic evaluations, consistent with the “value-based healthcare” paradigm [24–28] which is now emerging as the future methodology to decision-making.

### CEA model inputs

#### Healthcare resource consumption and costs

Nine-hundred and forty-two women responded to the e-survey and 700 of them reported a diagnosis of RAD. Respondents were 43 years old (range 26–69 years) and 179 underwent surgical intervention (84% open surgery). The mean time from the intervention was 605 days (range 44 days-12 years). Most of respondents were employees (37.1%) and housewives (20.6%).

A higher percentage of patients in the non-surgical group reported incontinence compared to women in the surgical group (58% vs. 14%). Similar figures were reported for lower back pain (81% vs. 13%). Table 1 shows data of problems related to RAD, while Table 2 summarizes the expenses sustained by the patients for the management of RAD.

Patients whose incontinence resolved without any intervention reported a mean total expense of 10.61€; patients who underwent RAD repair reported higher mean total costs in the range of 47.27-148.53€ depending upon the time from the intervention ( < = 1 year or > 1year). These higher costs are due to physiotherapy and postural exercises performed in the post-surgery period. In case of unresolved incontinence, the mean total cost for physiotherapy was 48.58€ in case of no surgical intervention and was in the range of 11.13–15.38€ for women who underwent RAD repair. In addition, patients with incontinence sustained monthly expenses for sanitary towels and physiotherapy of 10.25€ and in the range of 1.88–9.95€ for no surgical intervention and intervention groups, respectively.

**Table 1:** Statistics on problems related to RAD for the different groups of patients

RAD problems	No intervention		Intervention – time from the intervention < = 1 year		Intervention – time from the intervention > 1 year	
	N	%	N	%	N	%
**Incontinence**						
Not reported	4	1%		-		-
No	174	35%	25	27%	22	29%
Yes, I had incontinence problems but I solved them	28	6%	58	62%	42	55%
Yes, I still have incontinence problems	295	59%	11	12%	13	17%
**Lower-back pain**						
Not reported	46	9%	24	26%	17	22%
No	33	7%	16	17%	10	13%
Yes, I had lower-back pain but I solved them	18	4%	44	47%	39	51%
Yes, I still have lower-back pain	404	81%	10	11%	11	14%

Concerning lower-back pain, the mean total expense for physiotherapy or painkillers in case of resolved problems was 9€ for women who did not undergo the surgical intervention and was in the range of 198–212€ for women who underwent surgical repair of RAD. In cases of unresolved lower-back pain, the mean total expense for physiotherapy, acupuncture, osteopathy or physical exercises was 47.04€ in the non-surgical intervention group and ranged from 8.09€ to 25.97€ in the surgical intervention group. The monthly expense for physiotherapy and painkillers for the management of current lower-back pain was 26.50€ and 5.05–18.31€ for the surgical intervention group and non-intervention group, respectively.

Concerning total expenses for resolved or not resolved problems, for RAD repair strategy in the model we considered only costs reported for patients with time from the intervention < = 1 year to avoid double counting, considering that the values are similar in the two groups classified on time from RAD repair. These costs have been associated to RAD (first year) health state.

Regarding visits, the mean time lost for each visit was about 3–4 h across both groups. The mean number of visits related to RAD in the last 3 months was 0.79 in the group of patients who did not undergo surgical intervention; it was 1.60 for patients in the first year after the surgical repair and decreased to 0.28 in the following years after the intervention. Productivity losses were assessed according to the human capital approach [29]. Each working hour lost was quantified on the basis of the average hourly income associated with the professional category considered [30, 31]. In case of one day lost, 8 lost working hours were counted. Productivity losses are considered till retirement age (67 years in Italy [32]). Supplementary Table 2 shows the annual wages for the different professional figures.

The estimated monthly productivity loss for visits was 8.54€ in the non-intervention group and in the range 4.26–14.78€ for the intervention group, while the monthly expense for visits/exams, transportation, meals out of home was 47.37€ and 23.43-105.07€, respectively.

Patients in the first year after RAD repair reported higher productivity losses for the disease and higher expenses for paid assistance (186.89€ and 58.10€ per month, respectively).

**Table 2:** Summary of costs borne by the patients for the management of RAD

Cost item	No intervention	Intervention	
		Time from the intervention < = 1 year	Time from the intervention > 1 year
Mean total expense (sanitary towels, physiotherapy, physical exercises) for the management of incontinence (problems resolved)	10.53 €	148.53 €	47.27 €
Mean total expense (physiotherapy) for the management of incontinence (problems not resolved)	45.58 €	11.14 €	15.38 €
Mean total expense (physiotherapy, painkillers) for the management of lower-back pain (problems resolved)	9.10 €	198.10 €	212 €
Mean total expense (physiotherapy, acupuncture, osteopathy, physical exercises) for the management of lower-back pain (problems not resolved)	47.07 €	8.09 €	25.97 €
Monthly expense (physiotherapy, sanitary towels) for the management of incontinence (problems not resolved)	10.25 €	9.94 €	1.88 €
Monthly expense (physiotherapy, painkillers) for the management of lower-back pain (problems not resolved)	26.48 €	5.05 €	18.30 €
Monthly productivity loss for visits	8.54 €	14.78 €	4.26 €
Monthly expense for visits/exams, transportation, meals out of home	47.37 €	105.07 €	23.43 €
Monthly productivity loss for malaise	79.78 €	186.89 €	49.94 €
Monthly expense for paid assistance	48.35 €	58.10 €	34.21 €

Concerning NHS costs, for the group of patients who performed RAD repair, two specialist visits, before (code 89.7 A.4, 22€) and after the intervention (code 89.01.4, 16.20€) were considered according to the clinical practice reported by the clinicians involved in the study. Concerning RAD repair, we considered the cost for the laparoscopic intervention with mesh implantation and we applied in a conservative way the highest DRG reimbursement that may combine RAD repair with hernia repair (DRG 159, national tariff 4,892€).

The complications after RAD repair requiring procedural or surgical intervention, as assessed from the analysis of the “Italian Hernia Club” registry, were: 0.4% recurrences, 5.4% hematomas, 3.5% posterior rectus sheaths disruption, 3.1% seromas and 1.9% surgical site infections (superficial infections). NHS costs for the management of these complications have been retrieved from the literature in the Italian context [12]. Reported unit costs (€, 2017), uplifted to 2022, were: 4,058€ for recurrence (the cost is applied also for the management of posterior rectus sheaths disruption being the intervention similar), 334€ for surgical site infection (superficial infection) and 109€ for seroma/hematoma. By weighting these costs by the frequency of complications, we estimated a mean cost for complications management after RAD repair of 174€. We assumed that costs for the management of complications are sustained in the first year after RAD repair.

Supplementary Table 3 summarizes the parameters used to populate the CEA model with related sources and information on distributions applied in the probabilistic sensitivity analysis.

#### Quality of life

Patients who underwent RAD repair reported a substantial improvement in quality of life compared to women in the non-intervention group. The utility weight estimated from the EuroQol questionnaire responses was 0.87 in the first year and 0.91 in the subsequent years after the intervention versus 0.69 for patients who did not undergo the intervention (see Supplementary Table 3 for model input details).

#### Budget Impact analysis – BIA

A dynamic BIA model was developed to compare the standard of care scenario of management of women with RAD without surgical intervention with hypothetical future scenarios in which an increased adoption of the surgical repair of RAD from 2 to 10% will be considered, as for clinical advice, in the next 5 years (2% year 1, 4% year 2, 6% year 3, 8% year 4, 10% year 5). As surgical repair of RAD is reimbursed by the NHS in Italy only for those patients with important functional limitations, for the current scenario a 100% management of patients according to SOC has been considered.

In order to perform the BIA, a review of epidemiological data focused on women with RAD in Italy was carried out. Considering about 393,997 newborns in 2022 in Italy, a fecundity rate of 1.25 [33] (estimating 315,198 mothers), and taking into account that about one out of three women after pregnancy shows RAD [3, 4], we can estimate an incidence of about 105,000 women with RAD per year in Italy.

The cost of the current and new scenarios was determined by multiplying the yearly cost for each strategy by the proportion of the eligible population using it, considering subsequent yearly incident cohorts in order to obtain a dynamic model. Financial streams were presented as undiscounted costs, since the focus of the analysis was expected budget at each point [34]. The analysis has been integrated with the estimation of the impact of total QALYs gained.

## Results

### Cost-effectiveness analysis - CEA

**Fig. 2:**
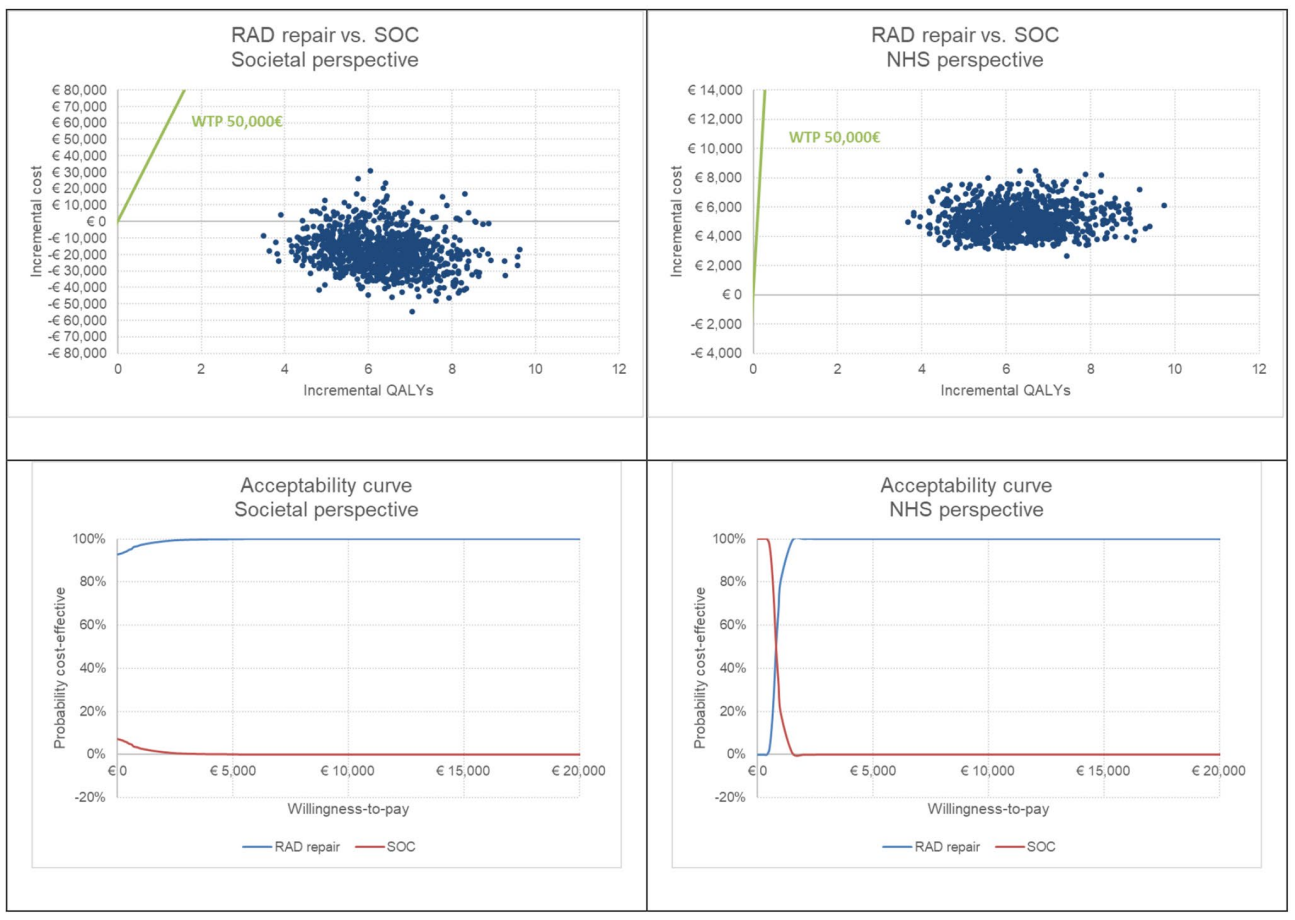
PSA results (cost-effectiveness planes and acceptability curves for the ICUR)

From the societal perspective, the model estimated over a lifetime horizon a mean cost of 64,115€ for a woman without RAD repair. On the other hand, a woman underwent surgical RAD repair showed a social cost of 46,541€. Estimated QALYs were 19.55 and 25.75 for the non-RAD repair group and RAD repair group, respectively. According to the results obtained, surgical repair of RAD may be considered a dominant option (less costly and with higher QALYs) compared to no intervention. Cost-effectiveness results are reported in Table 3. The probabilistic sensitivity analysis showed that RAD repair is a cost-effective choice in nearly all the simulations (Fig. 2, societal perspective). One-way sensitivity analyses confirmed the dominance of RAD repair for all parameters’ variations.

**Table 3:** Cost-effectiveness deterministic results

Perspective	Expected outcomes (discounted)	SOC	RAD repair	Difference	ICUR
Societal	Costs	€ 64,115	€ 46,541	-€ 17,575	Dominant
	QALYs	19.55	25.75	6.19	
NHS	Costs	€ 0	€ 5,104	€ 5,104	€ 824
	QALYs	19.55	25.75	6.19	

Considering the NHS perspective, the management of patients with RAD repair shows an additional cost of 5,104€ compared to SOC related to the intervention and to the specialist visits, leading to an ICUR of 824€/QALY. The probabilistic sensitivity analysis showed that RAD repair may be a cost-effective choice considering a WTP threshold higher than 2,000€/QALY (Fig. 2, NHS perspective). In the NHS perspective, one-way sensitivity analyses (Supplementary Fig. 2) showed that the cost for RAD repair and the utility weight for RAD repair for the subsequent years are the parameters most impacting the ICUR, which anyway has limited variations around the baseline result.

**Fig. 3:**
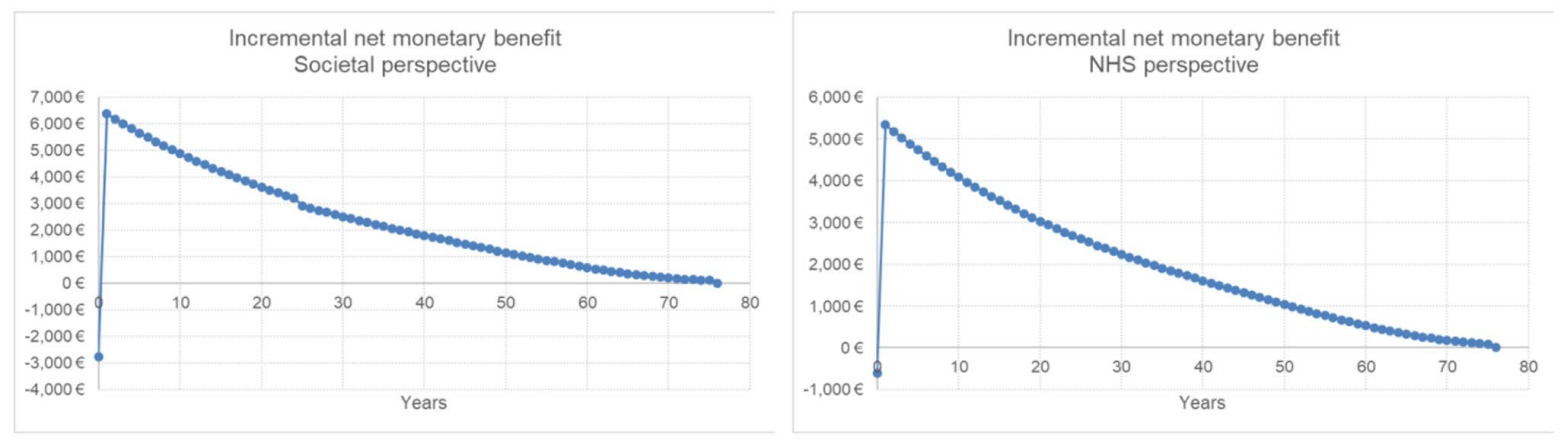
Incremental net monetary benefit of RAD repair compared to no intervention for societal and NHS perspectives (WTP = 25,000€/QALY) considering a lifetime horizon. For societal perspective, discontinuation in the graph is due to elimination of productivity losses after retirement age (67 years)

The analyses performed on INMB by applying a WTP threshold of 25,000€ per QALY showed that it starts to become positive the year after the intervention (year = 1) for both NHS and societal perspective indicating that the cost sustained by the NHS for RAD repair may be considered as an investment which is able to provide future greater benefits for the society as a whole (Fig. 3).

### Budget impact analysis

**Fig. 4:**
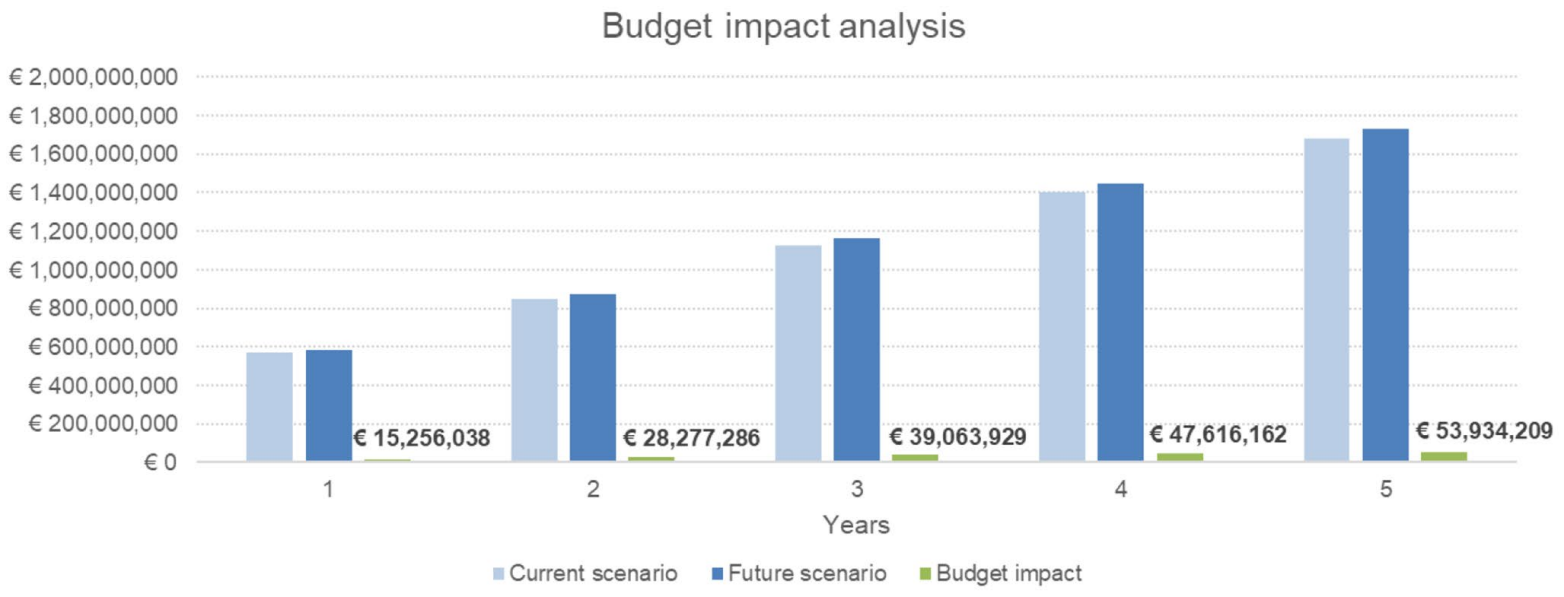
Representation of budget impact results

Compared to the current scenario of 100% SOC, the budget impact analysis from the societal perspective considering an increasing adoption of surgical RAD repair from 2 to 10% in the next 5 years (2% at year 1, 4% at year 2, 6% at year 3, 8% at year 4, 10% at year 5) shows an additional cost of 184,147,624€, corresponding to 351€ per patient. This cost is mainly (87% equal to 160,783,534€) sustained by the NHS. Anyway, this future scenario may lead to additional 16,155 total QALYs in the same period, equivalent to additional 0.03 QALYs per patient due to RAD repair. Table 4 reports the details of the budget impact analysis. Results are represented graphically in Fig. 4.

**Table 4:** Budget impact analysis from the societal perspective

Current scenario									
	SOC			Diastasis repair					
Year	% of patients	Users cohort	Cost	% of patients	Users cohort	Cost	**TOT budget impact**		
1	100%	105,000	€ 567,710,448	0%	0	€ 0	**€ 567,710,448**		
2	100%	105,000	€ 845,645,739	0%	0	€ 0	**€ 845,645,739**		
3	100%	105,000	€ 1,123,556,900	0%	0	€ 0	**€ 1,123,556,900**		
4	100%	105,000	€ 1,401,439,999	0%	0	€ 0	**€ 1,401,439,999**		
5	100%	105,000	€ 1,679,297,893	0%	0	€ 0	**€ 1,679,297,893**		
**Future scenario**									
	SOC			Diastasis repair					
Year	% of patients	Users cohort	Cost	% of patients	Users cohort	Cost	**TOT budget impact**	*Incremental expenses in comparison to current scenario*	
1	98%	102,900	€ 561,915,406	2%	2100	€ 21,051,080	**€ 582,966,486**	*€ 15,256,038*	
2	96%	100,800	€ 828,496,487	4%	4200	€ 45,426,537	**€ 873,923,025**	*€ 28,277,286*	
3	94%	98,700	€ 1,089,494,734	6%	6300	€ 73,126,095	**€ 1,162,620,829**	*€ 39,063,929*	
4	92%	96,600	€ 1,344,906,695	8%	8400	€ 104,149,466	**€ 1,449,056,161**	*€ 47,616,162*	
5	90%	94,500	€ 1,594,735,790	10%	10,500	€ 138,496,313	**€ 1,733,232,102**	*€ 53,934,209*	
**Total incremental result**									***€ 184,147,624***

## Discussion

Currently, in Italy there is no overall consensus on the surgical treatment for patients with post-pregnancy rectus diastasis and surgical repair is generally funded only in case of the presence of concomitant umbilical hernia or in case of extremely large diastasis and belly-bulging, causing frequent intertrigo and skin infections. In this context, minimally-invasive endo-laparoscopic RAD repair with mesh implant may be an option to be evaluated for these patients, taking into account the clinical benefits that emerged from the published literature [8, 35, 36]. Randomized-controlled trials (RCT) are in general considered the ones providing the highest level of evidence to demonstrate the efficacy of a new healthcare technology compared with an alternative, typically the standard of care. However, apart from the specific characteristics of technologies based on medical devices (e.g., small eligible patient population and recruitment, inability to blind clinicians and patients, choice of comparator group, and learning curve), the growing number of minimally invasive surgical techniques and different surgical specialties may make it difficult to perform a conventional RCT, and alternatives like real-world studies, have been proposed in order to minimize the impact of these problems [10, 11, 37, 38]. This is the case of this present study, in which we performed cost-effectiveness and budget impact analyses considering mini-invasive surgical repair of RAD versus no intervention from the NHS and societal perspectives in Italy based on real-world data.

Considering a lifetime horizon and the societal perspective, the analyses showed that mini-invasive RAD repair with the use of meshes may be a cost saving option compared to no intervention. Considering the young age of the patients, RAD repair may offer the immediate resolution of the disease with advantages, in terms of quality of life (and possibly return to working activities) prolonged over women’s entire lives. On the other hand, considering an increased adoption of the intervention from 2 to 10% in the next 5 years, the impact on the budget for the whole society shows an additional cost of about 184.1 million Euros but also about 16,200 additional QALYs. The additional cost at national level is due to the fact that it is only in the eighth year that RAD repair starts showing savings per patient compared to SOC (total cost of 21,102€ for RAD repair versus 21,285€ for SOC), but the savings did not emerge considering the short time horizon for the BIA.

Considering the NHS perspective, an additional cost emerges for RAD repair option, leading to an ICUR of 824€/QALY. Taking into account that in Italy, the willingness-to-pay thresholds used in these kind of studies range from 25,000€ to 74,700€ [20–22], RAD repair may be considered a cost-effective strategy also from the NHS perspective.

The analysis of the incremental net monetary benefit of RAD repair compared with SOC showed that positive values begin to occur in the first year after the intervention for both the perspectives considered. The study highlights that the NHS cost for RAD repair may be viewed as an investment able to provide future greater benefits for the society as a whole, allowing for a continuous improvement of the patients’ management process according to the value-based healthcare paradigm [25].

The current study presents few limitations that need to be highlighted. First of all, the analysis focused on the management of the main problems related to RAD, like incontinence and lower back pain, but often patients experience pelvic organ prolapse, intestinal problems and suffer from esthetic implications [36] which may decrease patients’ quality of life. Moreover, when incontinence is not recognized as a consequence of RAD, women may undergo laser therapy [39], urethral bulking agents [40] or tension-free tape procedure [41] that may be ineffective, thus further increasing the management costs in cases of no RAD repair. In addition, the respondents to the socio-economic questionnaire were not interviewed about aspects related to possible underlying hernia disease associated with RAD, which surely lowers patient’s quality of life and increases societal costs. Considering these observations, the results of our analysis may be considered conservative estimates of the possible advantages due to RAD repair compared to no intervention.

Secondly, the scenario analysis performed on the BIA model showed that results are mainly based on the assumptions on the future wider adoption of RAD repair, as estimated by clinicians; higher future utilization frequencies may lead to higher additional costs in the short term. A continuous monitoring and analysis of the adoption of the repair technique could give insights to better estimate present and future utilization rates.

Complication rates after surgical RAD repair were retrieved from a number of patients in a multicenter registry, while social costs were obtained through the administration of an online questionnaire and we do not know the level of overlapping between the two populations considered. In fact, the women who responded to the questionnaire mostly underwent traditional abdominoplasty (which is not minimally invasive and thus might lower the satisfaction rates concerning quality of life and aesthetics). The collection of data from the same population would have increased the internal consistency. On the other hand, patients’ characteristics were similar in the two subsets, starting from a patients’ age of 41 years in the registry compared to 43 years from the questionnaire; moreover, after RAD repair, the registry shows at 6 months 68% of patients with solved incontinence. This value is in line with the range 55-62% of patients who completed the questionnaire and that reported a solution of this problem after RAD repair.

Regarding the estimation of healthcare and non-healthcare resources from the societal perspective, it must be noted that data derived from self-reported questionnaires may be limited by varying recollection and poor generalizability. Variables collected from prospective observational multi-center studies would increase the validity of the current model. Observational studies would also allow evaluation of possible differences between types of meshes used in the surgical RAD repair. The analysis of the complications of RAD repair carried out on the “Italian Hernia Club” registry showed that innovative biosynthetic meshes are non-inferior to synthetic ones. From the surgeons’ perspective, these biosynthetic meshes might be the ideal meshes to be implanted in these cases (large population of young women who might in the future go through other pregnancies or other surgical operations), as soon as results from a long-term follow-up is available. Nevertheless, biosynthetic meshes, which fully resorb in 12–18 months, may lead to a lower risk of long-term complications related to the mesh [13, 42] and possibly improve the cost-effectiveness profile versus SOC. Further studies will be able to assess the sustainability of these innovative prostheses in the RAD context.

## Conclusions

In light of the lack of cost-effectiveness data for surgical RAD repair, the present study provides the first evidence about the clinical and economic advantages of the use of minimally invasive intervention with mesh implant in this context, showing that RAD repair compared to no intervention may be a cost-saving or a cost-effective option from the societal and NHS perspectives, respectively. Our study showed the advantages of RAD repair but highlighted also the need for further studies or registries, possibly involving different types of meshes and procedures. The questionnaire also raises the attention of the health providers on this current underestimated pathology, which deserves more attention as it represents a consistent health need. The validation of minimally-invasive approaches, as it happened in other surgical pathologies, carries substantial clinical advantages, and the present paper shows that these advantages are sustainable. Furthermore, this surgical approach leads to midline integrity restoration, a condition which allows also the application of long-term absorbable meshes; this combination results in promising outcomes and advantages especially in specific types of patients as fertile women and patients with long-life expectations. Those new techniques and materials should be included in an appropriate re-pay plan with an adequate DRG. In conclusion, these analyses provide evidences to move towards more innovative healthcare technologies for patients, according to the current harmonization process regarding the regulation on HTA at European level [43]. In the future, prospective randomized trials or registries, may provide a stronger level of recommendation. Ongoing and future analyses of the cost-effectiveness relationship accounting for different types of meshes, expense of materials, surgical procedures, potential complications and indirect costs would be greatly beneficial to clinicians and policy makers.

## Electronic supplementary material

Below is the link to the electronic supplementary material.

Supplementary Material 1

Supplementary Material 2

Supplementary Material 3

Supplementary Material 4

Supplementary Material 5

Supplementary Material 6

## Data Availability

The data that support the findings of this study are available from the corresponding author upon reasonable request.
