# Supplementary material for: Economic value of diastasis repair with the use of mesh compared to no intervention in Italy
Source: Eur J Health Econ. 2024 Mar 14;25(9):1569–80. doi: 10.1007/s10198-024-01685-z (PMC11512883; doi:10.1007/s10198-024-01685-z)
Supplement: Supplementary file 1 — Supplementary Material 1 [file 10198_2024_1685_MOESM1_ESM.docx]

APPENDIX 1

SOCIO-ECONOMIC QUESTIONNAIRE

Can you confirm that you have had a diagnosis of diastasis recti?

1. Yes

2. No

Please enter your date of birth: ………… (date)

Have you had repair surgery for diastasis recti?

1. Yes

2. No

If yes, please indicate the type of intervention:

1. Minimally invasive surgery (endoscopic/laparoscopic/robotic)

2. Traditional surgery (abdominoplasty)

Please indicate the date of the intervention (if you do not remember the precise date, at least enter the year and indicate January 1st): …….. (date)

EMPLOYMENT STATUS

What is your current employment status? (1 answer only)

Employee:

1. factory worker

2. employee

3. manager, director

Self-employed:

4. businesswoman, freelancer

5. other self-employed

Non-professional status:

6. retired

7. student

8. housewife

9. other, not employed

If you are a worker, what is your employment status? (1 answer only)

1. Full time

2. Part-time: 20 hours/week

3. Part-time: 24 hours/week

4. Part-time: 30 hours/week

5. Part-time: 36 hours/week

PATHOLOGY MANAGEMENT

Have you had or do you currently have incontinence problems?

1. No

2. Yes, I have had incontinence problems but have resolved them.

How long have you had incontinence? ..... months

If you used incontinence devices, please indicate how much you spent each month: ......€

How did you resolve it? (multiple answers possible)

□ I had surgery (e.g., sling, TVT, etc.)

□ I had physical therapy; Please indicate how much you spent: ......€

□ Other (specify)...............................; Indicate how much you spent: ......€

3. Yes. I still have incontinence problems.

How do you manage them? (multiple answers possible)

□ I have had surgery (e.g., sling, TVT, etc.) but it did not resolve the problems.

□ I do periodic physical therapy; Please indicate how much you spend per month: ......€

□ I use incontinence devices; Please indicate how much you spend per month: ......€

□ Other (specify)...............................; Indicate how much you spend per month: ......€

□ No management

Have you had or currently have low back pain?

1. No

2. Yes, I have had lower back pain but it has resolved.

How long have you had lower back pain? ..... months

How did you resolve it? (multiple answers possible)

□ I had physiotherapy or acupuncture; Indicate how much you spent: ......€

□ I have taken painkillers; Indicate how much you spent: ......€

□ Other (specify)...............................; Indicate how much you spent: ......€

3. Yes. I still have lower back pain.

How do you manage it? (multiple answers possible)

□ I periodically have physiotherapy or acupuncture; Please indicate how much you spend per month: ......€

□ I use painkillers; Please indicate how much you spend per month: ......€

□ Other (specify)...............................; Indicate how much you spend per month: ......€

□ No management

VISITS AND EXAMINATIONS

Have you had visits or examinations related to diastasis recti in the past 3 months? If yes, please also indicate the number.

1. No

2. Yes; Please specify the number of visits/exams: .............

If you had visits or examinations, how much time did you lose on average to have an exam or visit? Also take into account total travel time. (1 answer only)

1. About one hour

2. About two hours

3. From 2 to 4 hours

4. From 4 to 6 hours

5. One day

If you have had visits or examinations, please indicate how much you spent on average to have an examination or visit (also take into account the cost of any accompanying person).

1. Cost of the visit or examination (e.g., ticket or amount if performed privately): ..........€

2. Transportation costs (e.g., bus, streetcar, metro, train, cab, parking, fuel, tolls): ..........€

3. Expenses for meals away from home: ..........€

4. Other expenses (please specify).......................................... : ..........€

**FORMAL ASSISTANCE**

In the past month, because of issues related to diastasis recti (e.g., lower back pain), have you turned to paid contractors/workers for household help (e.g., babysitter, domestic helper)? (1 answer only)

1. No

2. Yes; How much did you spend? ...... €

LIMITATIONS CAUSED BY THE PATHOLOGY

In the past 3 months, approximately how many days of work (professional or home) have you lost due to problems related to diastasis recti (if half a day, indicate 0.5)? Exclude any days you have missed for visits or examinations.

.... days

In the past 3 months, how many days have you missed out on activities related to your personal and social life (e.g., going out with friends, hobbies, sports, family activities, etc.) due to problems related to diastasis recti (if half a day, indicate 0.5)?

.... days
