# Supplementary material for: Economic value of diastasis repair with the use of mesh compared to no intervention in Italy
Source: Eur J Health Econ. 2024 Mar 14;25(9):1569–80. doi: 10.1007/s10198-024-01685-z (PMC11512883; doi:10.1007/s10198-024-01685-z)
Supplement: Supplementary file 3 — Supplementary Material 3 [file 10198_2024_1685_MOESM3_ESM.docx]

Supplementary Table 1 – Transition probabilities to death state

| **Age (years)** | **Yearly mortality**  **(females)** |
| --- | --- |
| 43 | 0.0764% |
| 44 | 0.0858% |
| 45 | 0.0930% |
| 46 | 0.1031% |
| 47 | 0.1132% |
| 48 | 0.1213% |
| 49 | 0.1348% |
| 50 | 0.1495% |
| 51 | 0.1664% |
| 52 | 0.1870% |
| 53 | 0.2040% |
| 54 | 0.2210% |
| 55 | 0.2386% |
| 56 | 0.2541% |
| 57 | 0.2812% |
| 58 | 0.3125% |
| 59 | 0.3448% |
| 60 | 0.3809% |
| 61 | 0.4202% |
| 62 | 0.4614% |
| 63 | 0.5058% |
| 64 | 0.5526% |
| 65 | 0.6092% |
| 66 | 0.6698% |
| 67 | 0.7469% |
| 68 | 0.8358% |
| 69 | 0.9122% |
| 70 | 1.0202% |
| 71 | 1.1429% |
| 72 | 1.2915% |
| 73 | 1.3936% |
| 74 | 1.5289% |
| 75 | 1.7139% |
| 76 | 1.9706% |
| 77 | 2.2401% |
| 78 | 2.6246% |
| 79 | 2.9508% |
| 80 | 3.3270% |
| 81 | 3.7826% |
| 82 | 4.2478% |
| 83 | 4.9074% |
| 84 | 5.7296% |
| 85 | 6.6770% |
| 86 | 7.7546% |
| 87 | 8.8908% |
| 88 | 10.2902% |
| 89 | 11.7530% |
| 90 | 13.1830% |
| 91 | 14.9466% |
| 92 | 16.6968% |
| 93 | 18.7141% |
| 94 | 20.8684% |
| 95 | 23.0894% |
| 96 | 25.0665% |
| 97 | 27.3383% |
| 98 | 29.8107% |
| 99 | 32.5607% |
| 100 | 35.5882% |
| 101 | 38.8754% |
| 102 | 42.0372% |
| 103 | 45.2666% |
| 104 | 48.5372% |
| 105 | 51.8213% |
| 106 | 55.0910% |
| 107 | 58.3182% |
| 108 | 61.4768% |
| 109 | 64.5430% |
| 110 | 67.4954% |
| 111 | 70.3162% |
| 112 | 72.9917% |
| 113 | 75.5114% |
| 114 | 77.8686% |
| 115 | 80.0602% |
| 116 | 82.0860% |
| 117 | 83.9485% |
| 118 | 85.6525% |
| 119 | 87.2042% |
