# Supplementary material for: Economic value of diastasis repair with the use of mesh compared to no intervention in Italy
Source: Eur J Health Econ. 2024 Mar 14;25(9):1569–80. doi: 10.1007/s10198-024-01685-z (PMC11512883; doi:10.1007/s10198-024-01685-z)
Supplement: Supplementary file 4 — Supplementary Material 4 [file 10198_2024_1685_MOESM4_ESM.docx]

Supplementary Table 2 - Annual wages for the different professional figures

| **Profession** | **Annual wage** | **Reference** |
| --- | --- | --- |
| Blue-collar worker | 17,790€ | [30] |
| Office worker | 24,215€ |  |
| Manager, executive | 60,615€ |  |
| Business-owner, member of profession | 54,171€ |  |
| Other self-employed | 25,154€ |  |
| Housewife | 11,592€ | [31] |
