# Supplementary material for: Economic value of diastasis repair with the use of mesh compared to no intervention in Italy
Source: Eur J Health Econ. 2024 Mar 14;25(9):1569–80. doi: 10.1007/s10198-024-01685-z (PMC11512883; doi:10.1007/s10198-024-01685-z)
Supplement: Supplementary file 5 — Supplementary Material 5 [file 10198_2024_1685_MOESM5_ESM.docx]

Supplementary Table 3 – Model input parameters with related distributions for the probabilistic sensitivity analysis

| **Parameter** | **Baseline value** | **Standard error of the mean** | **Distribution** | **Alpha/ mean of logs** | **Beta/lambda/ standard error of logs** | **Reference** |
| --- | --- | --- | --- | --- | --- | --- |
| **Standard of care** | | | | | | |
| Mean n. months incontinence (incontinence solved) | 13.560 | 4.260* | Gamma | 10.132 | 1.338 | SEQ |
| Mean monthly cost incontinence devices (incontinence solved) | 13.120 | 3.770 | Gamma | 12.111 | 1.083 | SEQ |
| % pts reporting use of incontinence devices (incontinence solved) | 0.016 | 0.002* | Beta | 94.487 | 5810.973 | SEQ |
| Mean total cost physiotherapy/exercises (incontinence solved) | 214.000 | 51.650 | Gamma | 17.167 | 12.466 | SEQ |
| % pts reporting physiotherapy/exercises (incontinence solved) | 0.036 | 0.004* | Beta | 92.556 | 2485.613 | SEQ |
| Mean monthly cost management incontinence (incontinence not solved) | 39.200 | 5.590 | Gamma | 49.175 | 0.797 | SEQ |
| % pts reporting management incontinence (incontinence not solved) | 0.262 | 0.027* | Beta | 70.664 | 199.562 | SEQ |
| Mean total cost physiotherapy (incontinence not solved) | 557.350 | 110.960 | Gamma | 25.230 | 22.090 | SEQ |
| % pts reporting physiotherapy (incontinence not solved) | 0.082 | 0.008* | Beta | 88.102 | 988.941 | SEQ |
| Mean total cost lower-back pain management (pain solved) | 351.460 | 94.010 | Gamma | 13.977 | 25.146 | SEQ |
| % pts reporting lower-back pain management (pain solved) | 0.026 | 0.003* | Beta | 93.527 | 3517.541 | SEQ |
| Mean monthly cost management lower-back pain (pain not solved) | 60.590 | 6.030 | Gamma | 100.964 | 0.600 | SEQ |
| % pts reporting management lower-back pain (pain not solved) | 0.437 | 0.045* | Beta | 53.624 | 69.057 | SEQ |
| Mean total cost management lower-back pain (pain not solved) | 491.350 | 68.550 | Gamma | 51.377 | 9.564 | SEQ |
| % pts reporting lower-back pain (pain not solved) | 0.096 | 0.010* | Beta | 86.744 | 818.722 | SEQ |
| Mean n. hours lost per visit | 3.110 | 0.080 | Gamma | 1511.266 | 0.002 | SEQ |
| Mean n. visit/3 months | 0.760 | 0.050 | Gamma | 231.040 | 0.003 | SEQ |
| Mean hourly wage | 10.840 | 0.310 | Gamma | 1222.743 | 0.009 | SEQ, [30,31] |
| Mean working days lost for malaise/3 months | 2.760 | 0.360 | Gamma | 58.778 | 0.047 | SEQ |
| Mean OOP cost per visit | 187.000 | 4.310 | Gamma | 1882.473 | 0.099 | SEQ |
| Mean monthly expense for paid assistance | 48.350 | 7.970 | Gamma | 36.802 | 1.314 | SEQ |
| **RAD repair** | | | | | | |
| Cost visits pre and post intervention | 38.200 | 7.600 | Gamma | 25.000 | 1.520 | codes 89.7A.4 and 89.01.4,  National price list |
| Cost RAD repair | 4892.000 | 978.400 | Gamma | 25.000 | 195.680 | DRG 159, national tariff |
| Cost management of diastasis repair complications | 174.039 | 34.808 | Gamma | 25.000 | 6.962 | “Italian Hernia Club” registry, [12] |
| **RAD (first year)** | | | | | | |
| Mean n. months incontinence (incontinence solved) | 35.820 | 4.26* | Gamma | 70.70 | 0.51 | SEQ |
| Mean monthly cost incontinence devices (incontinence solved) | 17.00 | 3.77 | Gamma | 20.33 | 0.84 | SEQ |
| % pts reporting use of incontinence devices (incontinence solved) | 0.1596 | 0.016* | Beta | 80.55 | 424.16 | SEQ |
| Mean total cost physiotherapy/exercises (incontinence solved) | 254.050 | 65.974 | Gamma | 14.828 | 17.133 | SEQ |
| % pts reporting physiotherapy/exercises (incontinence solved) | 0.202 | 0.021* | Beta | 76.428 | 301.742 | SEQ |
| Mean monthly cost management incontinence (incontinence not solved) | 133.430 | 61.300 | Gamma | 4.738 | 28.162 | SEQ |
| % pts reporting management incontinence (incontinence not solved) | 0.075 | 0.008* | Beta | 88.811 | 1103.277 | SEQ |
| Mean total cost physiotherapy (incontinence not solved) | 523.080 | 476.925 | Gamma | 1.203 | 434.843 | SEQ |
| % pts reporting physiotherapy (incontinence not solved) | 0.021 | 0.002* | Beta | 93.973 | 4317.907 | SEQ |
| Mean total cost lower-back pain management (pain solved) | 503.300 | 152.986 | Gamma | 10.823 | 46.502 | SEQ |
| % pts reporting lower-back pain management (pain solved) | 0.394 | 0.040* | Beta | 57.845 | 89.119 | SEQ |
| Mean monthly cost management lower-back pain (pain not solved) | 79.170 | 34.800 | Gamma | 5.176 | 15.297 | SEQ |
| % pts reporting management lower-back pain (pain not solved) | 0.064 | 0.007* | Beta | 89.849 | 1318.440 | SEQ |
| Mean total cost management lower-back pain (pain not solved) | 380.000 | 120.000 | Gamma | 10.028 | 37.895 | SEQ |
| % pts reporting management lower-back pain (pain not solved) | 0.021 | 0.002* | Beta | 93.973 | 4317.907 | SEQ |
| Mean n. hours lost per visit | 2.590 | 0.180 | Gamma | 207.040 | 0.013 | SEQ |
| Mean n. visit/3 months | 1.600 | 0.220 | Gamma | 52.893 | 0.030 | SEQ |
| Mean hourly wage | 10.700 | 0.710 | Gamma | 227.118 | 0.047 | SEQ, [30,31] |
| Mean working days lost for malaise/3 months | 6.550 | 2.120 | Gamma | 9.546 | 0.686 | SEQ |
| Mean OOP cost per visit | 197.000 | 7.370 | Gamma | 714.492 | 0.276 | SEQ |
| Mean monthly expense for paid assistance | 58.100 | 25.480 | Gamma | 5.199 | 11.174 | SEQ |
| **RAD (subsequent years)** | | | | | | |
| Mean monthly cost management incontinence (incontinence not solved) | 36.250 | 22.490 | Gamma | 2.598 | 13.953 | SEQ |
| % pts reporting management incontinence (incontinence not solved) | 0.052 | 0.005* | Beta | 91.004 | 1662.438 | SEQ |
| Mean monthly cost management lower-back pain (pain not solved) | 282.000 | 375.558 | Gamma | 0.564 | 500.156 | SEQ |
| % pts reporting management lower-back pain (pain not solved) | 0.065 | 0.007* | Beta | 89.742 | 1293.033 | SEQ |
| Mean n. hours lost per visit | 4.000 | 0.210 | Gamma | 362.812 | 0.011 | SEQ |
| Mean n. visit/3 months | 0.280 | 0.110 | Gamma | 6.479 | 0.043 | SEQ |
| Mean hourly wage | 11.420 | 0.960 | Gamma | 141.511 | 0.081 | SEQ, [30,31] |
| Mean working days lost for malaise/3 months | 1.640 | 1.240 | Gamma | 1.749 | 0.938 | SEQ |
| Mean OOP cost per visit | 251.000 | 13.160 | Gamma | 363.777 | 0.690 | SEQ |
| Mean monthly expense for paid assistance | 34.210 | 26.743 | Gamma | 1.636 | 20.905 | SEQ |
| **Utilities** |  |  |  |  |  |  |
| u SOC | 0.690 | 0.010 | Beta | 1475.220 | 662.780 | EQ |
| u RAD repair (first year) | 0.870 | 0.010 | Beta | 983.100 | 146.900 | EQ |
| u RAD repair (second year onwards) | 0.910 | 0.020 | Beta | 185.413 | 18.338 | EQ |

* Variation of ±20% of the basal value; pts=patients; SEQ=socio-economic questionnaire; EQ=EuroQol 5D 5L
