# Supplementary figures and images for: Economic value of diastasis repair with the use of mesh compared to no intervention in Italy
Source: Eur J Health Econ. 2024 Mar 14;25(9):1569–80. doi: 10.1007/s10198-024-01685-z (PMC11512883; doi:10.1007/s10198-024-01685-z)

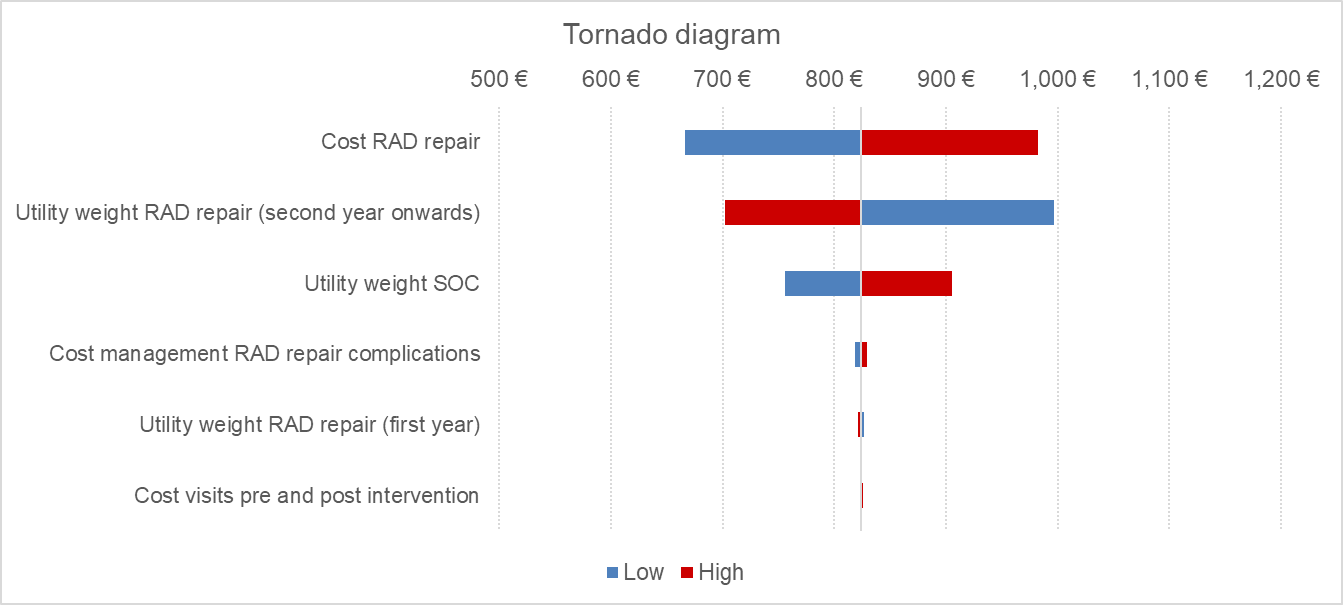


Supplementary Figure 1 – Deterministic sensitivity analyses for the ICUR (NHS perspective)

Supplement: Supplementary file 6 — Supplementary Material 6 [file 10198_2024_1685_MOESM6_ESM.docx]
